# First report of the Asian tapeworm *Schyzocotyle acheilognathi* in the Colombian freshwater fish *Parodon magdalenensis*

**DOI:** 10.3389/fvets.2025.1614531

**Published:** 2025-07-09

**Authors:** Astrid Rave, Sara López-Osorio, Manuel Uribe, Carlos Hermosilla, Jenny J. Chaparro-Gutiérrez

**Affiliations:** ^1^CIBAV Research Group, Veterinary Medicine School, Universidad de Antioquia, Medellín, Colombia; ^2^Biomedical Research Center Seltersberg (BFS), Institute of Parasitology, Justus Liebig University Giessen, Giessen, Germany

**Keywords:** Asian tapeworm, *Parodon magdalenensis*, native fish, freshwater fish, neotropical

## Abstract

The Asian fish tapeworm (AFT), scientifically known as *Schyzocotyle acheilognathi*, is a commonly encountered invasive parasite that has great relevance in freshwater ecosystems. This euryxenous cestode has a complex life cycle and includes a wide range of definitive hosts. It has also been reported in more than 300 species of free-living and farmed fish, amphibians, reptiles, and birds. Its remarkable pathogenicity leads to high mortality rates in infected fish, particularly among cyprinids. The environmental adaptability of *S. acheilognathi* has contributed to its global spread across continents, excluding Antarctica. In South America, *S. acheilognathi* has been reported in Brazil and Argentina. In this study, we examined 103 specimens of *Parodon magdalenensis*, a hyperendemic characiform fish species native to the Magdalena River basin in Colombia, and found a parasite prevalence in 32 of 103 specimens (31.07%). This study presents the first morphological and molecular characterization of the AFT in Colombia and its identification in a new host species (*P. magdalenensis*), supported by rDNA sequences from the 28S, 18S, and 16S regions.

## 1 Introduction

*Schyzocotyle acheilognathi,* known as the Asian fish tapeworm (AFT), was first identified in the intestine of *Acheilognathus rhombeus* from Lake Ogura in Japan (1). Since its initial discovery, the taxonomic classification of this species has remained complex. It has been assigned various synonymous names, likely due to the presence of conspecific cestodes that share a characteristic heart- or arrow-shaped scolex with narrow, deep attachment grooves known as bothria. These morphological traits are also common to the genus *Clestobothrium* within the family Bothriocephalidae. These morphological similarities have led to the description of different species across various genera, hosts, and regions (2), including *Botriocephalus opsalichthydis, B. fluviatilis, B. gowkongensi, B.* (*Clestobothrium*) *kivuensis, B. sinensis, B. phoxini, B. aegyptiacus, Ptychobothrium chelai, P. khami, P. duscusae, P. phuloi, P. rojanapaibuli, Capoorai barilii, and Coelobothrium oitense* (2–7). The most commonly used name is *Bothriocephalus acheilognathi*; however, a comprehensive molecular phylogenetic analysis of the family Bothriocephalidae indicated that this species belongs to a separate genus, *S. acheilognathi* (8).

The cestode species *Schyzocotyle acheilognathi* is a parasite native to East Asia that has recently spread extensively across all continents except Antarctica (9). This spread is mainly attributed to the import of common carp (*Cyprinus carpio*) and grass carp (*Ctenopharyngodon idella*) for aquaculture proposes (2). Moreover, the unregulated trade of ornamental fish and minnows (families Cyprinidae and Leuciscidae), often used as bait, has contributed to the rapid worldwide dispersion of *Schyzocotyle acheilognathi* (10, 11). Since its first report in Japan, *S. acheilognathi* has spread to various countries in Asia, initially reaching China (3) and Sri Lanka in 1960, where it was identified as *B. gowkongensis* in *Systomus sarana* (12). It was later documented in India, Korea, Nepal, Malaysia, the Philippines, Thailand, Turkey, and Uzbekistan (13–20). In Europe, *S. acheilognathi* was first recorded in *Cyprinus carpio* in Ukraine (21), followed by its spread across nearly the entire continent (2). In North America, the first record dates back to 1975 in the United States (Arkansas), where it was found in two cyprinid species, *Notemigonus crysoleucas* and *Pimephales promelas*. It is suggested that the introduction of the alien species *S. acheilognathi* in the continent probably occurred through the importation of grass carp (*Ctenopharyngodon idella*) from Malaysia and Taiwan in 1963 (22). Subsequently, its presence was reported in other places such as Mexico, Canada (16), and Central America, specifically in Guatemala, Honduras, and Panama (23, 24). In South America, the distribution of the AFT is limited to Patagonia in Argentina and Brazil, specifically in the species *Cyprinus carpio* (25, 26). However, a recent study has documented the first record of the AFT in a native Brazilian species, the scaleless *Rineloricaria pentamaculata* (Siluriformes) (27).

**Figure 1:**
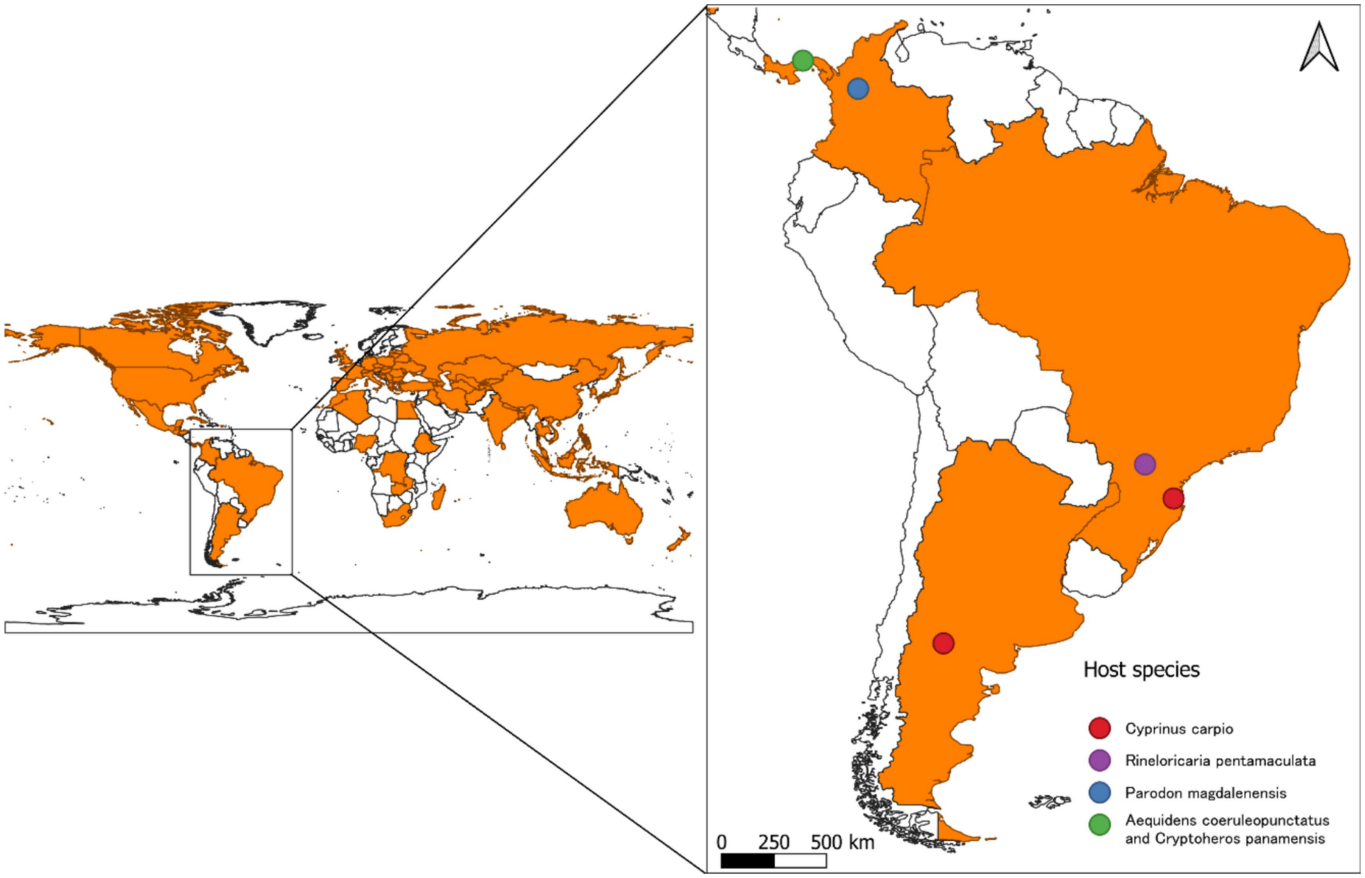
Global distribution map of *Schyzocotyle acheilognathi.*

Since its initial identification, *S. acheilognathi* has been recognized as a significant pathogen in Asian and European fisheries (28), causing considerable economic losses of captive-raised juvenile fish (29, 30). The host fish species of the AFT are distributed across tropical, subtropical, and temperate regions. The cestode species *S. acheilognathi* is considered thermophilic, with an optimal reproduction temperature ranging from 25 to 30°C (31). However, it has also been reported in areas with lower temperatures, where development occurs notably slower below 12°C (2) (Figure 1).

**Figure 2:**
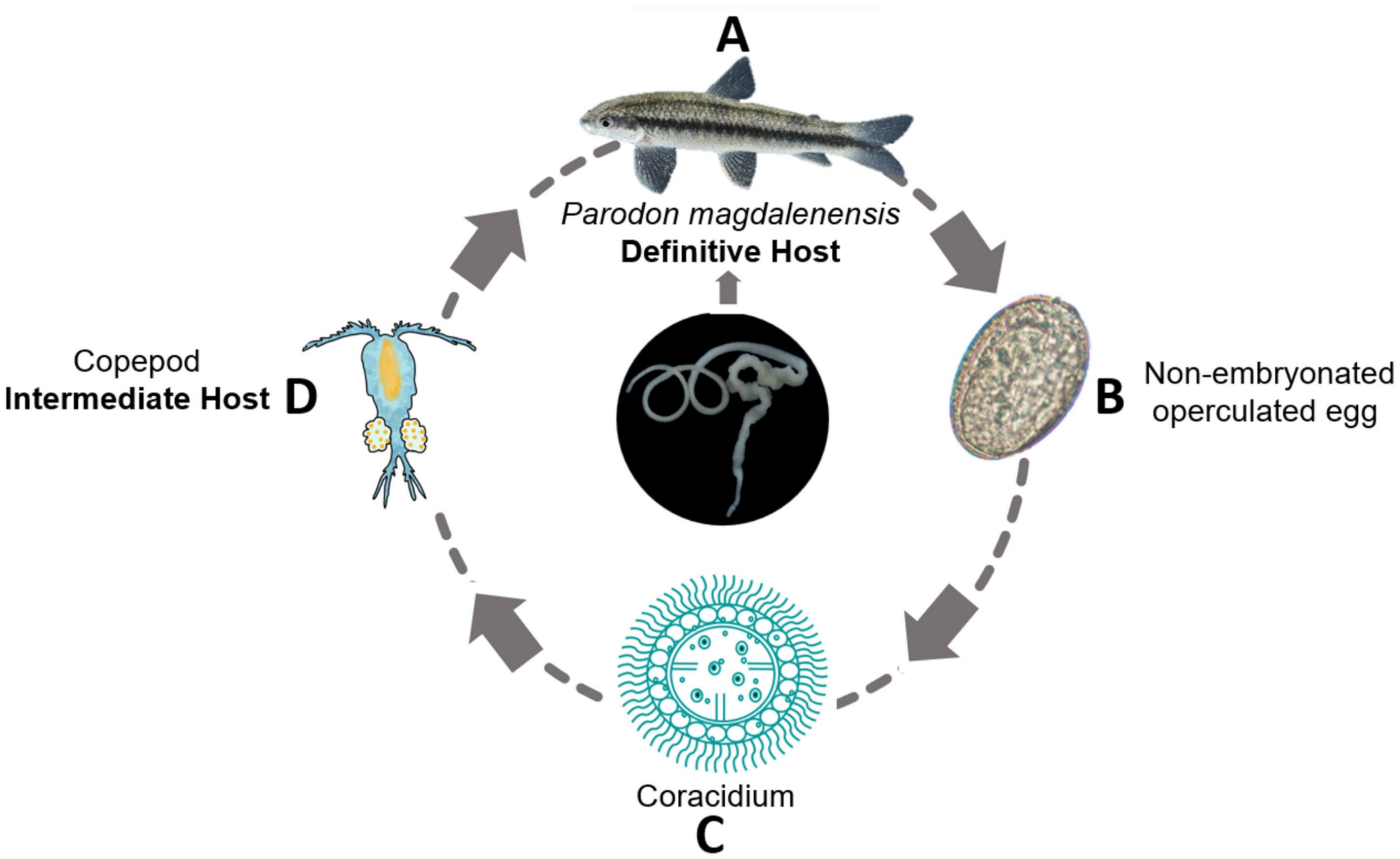
The life cycle of the Asian fish tapeworm (*Schyzocotyle acheilognathi*). Adult stages of the parasite are commonly located in the intestines of freshwater fish, which serve as the main definitive host **(A)**. Mature proglottids contain and subsequently release non-embryonated, operculated eggs through feces into the water **(B)**. Inside the eggs, the hexacanth embryo develops, enveloped by a ciliated embryophore called a coracidium **(C)**. After being ingested by copepods, the coracidium develops into a larval form known as the plerocercoid **(D)**. The cycle is completed when freshwater fish ingest copepods containing plerocercoids.

The AFT follows a simple bothriocephaliid heteroxenus life cycle involving two hosts (Figure 2): planktonic copepods (Copepoda: Cyclopidae) as intermediate hosts and freshwater fish as definitive hosts. A diverse range of fish species has been reported as definitive hosts (2), with the number increasing over recent decades (32). In addition, the AFT has been documented in other vertebrates, including amphibians, reptiles, and birds. There have also been reports of parasite eggs in human feces (33). However, these cases should be interpreted with caution as they might have resulted from post-cyclic or accidental transmission (2).

The life cycle of *S. acheilognathi* is completed in approximately one month. The adult parasite attaches to the intestine of freshwater fish and releases non-embryonated, operculated eggs into the water through feces. Depending on the water temperature, within a few days, an embryo (oncosphere or six-hooked hexacanth) forms inside the egg (9). The hexacanth embryo is enveloped within a ciliated embryophore called a coracidium, whose surrounding ciliated cells enable active movement in water until it is ingested by copepods of the genera *Acanthocyclops* sp., *Cyclops* sp., *Macrocyclops* sp., *Megacyclops* sp., *Mesocyclops* sp., *Thermocyclops* sp., and *Tropocyclops* sp. (34). Upon ingestion by copepods, the coracidium sheds its ciliated cells, penetrates the gut into the coelom, and develops into a larval stage called plerocercoid. The duration of plerocercoid larvae development depends on water temperature, taking approximately 21–23 days at 28–29°C and approximately 1.5–2 months at lower temperatures of 15–22°C (31).

The life cycle continues when fish ingest copepods infected with plerocercoids. Once inside the fish’s intestine, the plerocercoids attach to the intestine wall and complete their development into the adult stage, producing eggs over a period of approximately 20 days (31).

## 2 Methodology

### 2.1 Study area and sample collection

The Porce River is part of the Magdalena River Basin, which is the largest river system in the northern Andean region of Colombia. It originates at an elevation of 2,660 meters above the sea level, travels 2,247 km to the north, joins the Nechí River at an elevation of 170 meters above the sea level, then merges with the Cauca River, and finally joins the Magdalena River before flowing into the Caribbean Sea. This basin is of critical importance to Colombia, encompassing 84% of the hydropower plants that supply energy to the country (35). The two hydropower plants that are part of the Magdalena-Cauca basin are the Porce II and Porce III hydropower plants, owned by Empresas Públicas de Medellín. They are located in the northeastern region of the Antioquia department (36).

**Figure 3:**
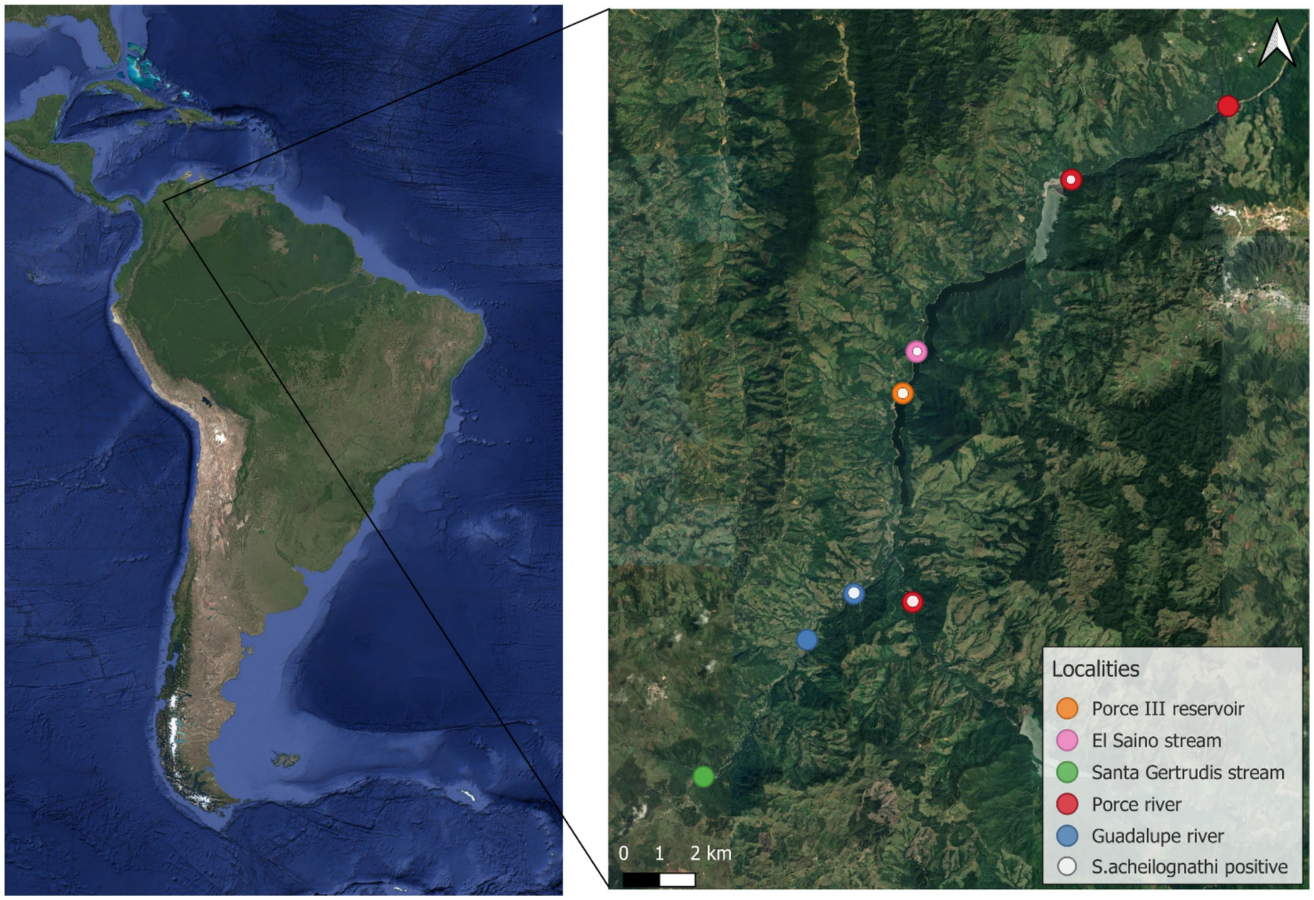
Precise geographic location of the sampling area.

A total of 103 specimens of *Parodon magdalenensis* were collected from 8 localities within the area influenced by the Porce II and Porce III dams between May 2022 and April 2023 (Supplementary Table 1), (Figure 3). The fish were captured in rivers and streams using cast nets with variable mesh sizes (0.5, 1.5, and 2.0 cm) and a portable electrofishing device with pulsed current of 1 ampere (340 V, 1–2 A, DC). In the dam areas, the capture method involved the use of gill nets and seine nets.

An incision was made using Metzenbaum scissors, starting from the caudal region of the pectoral fin, ascending toward the midline, and descending toward the anus to create a necropsy window for observing the internal organs (37). These organs were examined in a petri dish under a Nikon SMZ-1 stereomicroscope to search for parasites (Nikon). The metazoan parasites found were carefully recovered from the intestine.

### 2.2 Morphological analysis

The parasites recovered during the fish necropsy procedures were fixed in 10% formalin and preserved in 70% ethanol for morphological identification in accordance with previous studies (25, 32). In addition, the helminths collected from the intestine of *P. magdalenensis* were fixed in 96% ethanol until further molecular analysis (38).

General morphological and morphometric taxonomic traits were observed, and the parasites were stained with acetic carmine and mounted in Canada Balsam (39). The proglottids containing the eggs were dissected onto microscope slides using a saline solution for egg observation.

Specimen identification was conducted based on overall morphology, including the shape and size of the scolex and proglottid (40, 41). Photographs were taken using an Olympus BX53 microscope (Olympus Corporation, Tokyo, Japan) (100x, 400x, and 1,000x), equipped with an Olympus DP74 digital camera (Olympus Corporation, Tokyo, Japan), and the Olympus SZXY stereomicroscope (Olympus Corporation, Tokyo, Japan), equipped with an Olympus DP27 digital camera. These instruments utilized Olympus cellSens Standard software (Olympus Corporation, Tokyo, Japan) for analysis.

### 2.3 Molecular analysis

One sequence of each gene (ssrDNA, lsrDNA, nuclear rDNA) was *de novo* sequenced, and parasite specimens were obtained from freshly sacrificed fish and fixed in molecular-grade ethanol at 96–99% according to the procedure described by a previous study (38). Total genomic DNA was extracted using the GeneJet Genomic DNA Purification Kit (Thermo Scientific, Waltham, United States), following the manufacturer’s instructions. The PCR protocols, primer usage, and PCR purification steps followed the methodology described in a previous study (42). PCR products were analyzed on a 1% agarose electrophoresis gel and bidirectionally sequenced using Sanger technology at a commercial laboratory (Macrogen, South Korea). Contiguous sequences of complete ssrDNA (18S RNA) (1,316 bp), partial lsrDNA (28S RNA) (domains D1-D3; 1,576 bp), and partial rrnL (16S RNA) (720 bp) were assembled using Geneious Prime 2023.2.1 software and Mega11 (Molecular Evolutionary Genetics Analysis), and sequence identity was checked with the Basic Local Alignment Search Tool (BLAST).^1^ Phylogenetic relationships were estimated from individual genes and from the concatenated dataset using maximum likelihood (ML) and Bayesian inference (BI) methods. The phylogenetic tree was constructed using the maximum likelihood criterion in IQ-TREE (43, 44). The nucleotide evolution model that best fit the data was TIMe+G4. Nodal support values were estimated by running 5,000 replicates with standard non-parametric bootstrap support (1,000 repetitions). A genetic algorithm approach was used for the phylogenetic analysis of large biological sequence datasets under the maximum likelihood criterion. A phylogenetic tree was constructed using the ssrDNA (18S RNA), partial lsrDNA (28S RNA) (domains D1-D3), and partial rrnL (16S RNA) sequences from species of the genera *Bothriocephalus* sp. and *Schyzocotyle* sp. available in the GenBank database (Supplementary Table 2). A sequence from *Grillotia* sp. (Cestoda: Trypanorhyncha) was used as the outgroup taxon.

### 2.4 Data Accessibility

The parasites isolated from *Parodon magdalenensis* were deposited in the Parasitology Museum collection at Universidad de Antioquia, Medellín, Colombia, under the occurrence ID 177-PA-FCA-UdeA. The nucleotide sequences isolated from *Schyzocotyle acheilognathi* reported in this study are available in the GenBank database under accession numbers PQ530045, PQ528318, and PQ516284.

## 3 Results

### 3.1 Morphological identification

**Figure 4:**
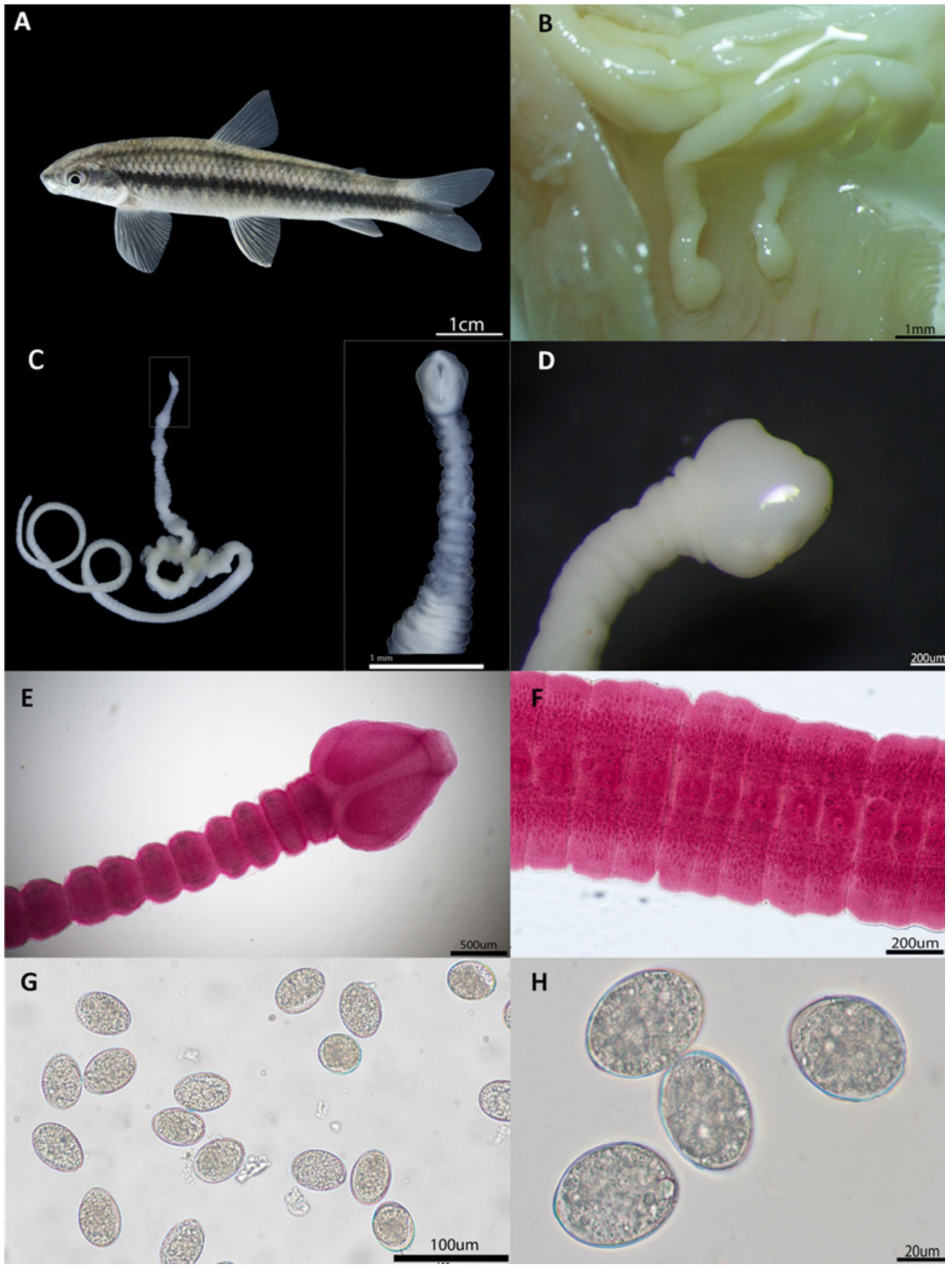
Macro- and micro-photographs of different parasite stages of *Schyzocotyle acheilognathi* in **(A)**. the newly reported definitive host *P. magdalenensis*. **(B)** Adult stages of *S. acheilognathi* firmly attached to the small intestine of the definitive host. **(C)** Whole parasite and side-view close-up of the scolex. **(D,E)** Classical arrow-shaped scolex. **(F)**. Mature proglottids with a distinctive genital pore. **(G,H)** Non-embryonated operculated eggs.

Helminths were found in 32 of the 103 (31.07%) *Parodon magdalenensis* specimens, with cestodes morphologically compatible with *S. acheilognathi* distributed throughout the intestine. The cestodes recovered from the intestine of *P. magdalenensis* (Figure 4A) measured 70.14 mm ± 31.32 in length and 1.4 mm ± 0.41 in width. They composed of numerous mature proglottids, measuring 0.177 mm ± 0.067 in length and 1.147 mm ± 0.029 in width, and gravid proglottids, measuring 0.224 mm ± 0.037 in width. They displayed a whitish color (Figures 4B–D) and a flat, elongated body composed of numerous proglottids separated from each other. They exhibited a hookless scolex with a heart- or arrow-shaped, nearly spherical form. The apical disc was weakly developed and unarmed, featuring two short, deep bothria and characterized by small openings positioned dorsally and ventrally. The neck was absent. The initial proglottids following the scolex were considerably narrower than the scolex itself (Figure 4E). Medullary testes were observed to be spherical to oval, and the bilobed ovary was located near the posterior margin of the proglottid. Cortical vitelline follicles, ranging from spherical to oval, were present and formed two lateral bands along the length of the proglottids (Figure 4F). The operculated, unembryonated eggs measured 0.046 ± 0.056 mm in length and 0.031 ± 0.036 mm in width (Figures 4G,H).

### 3.2 Molecular identification

**Figure 5:**
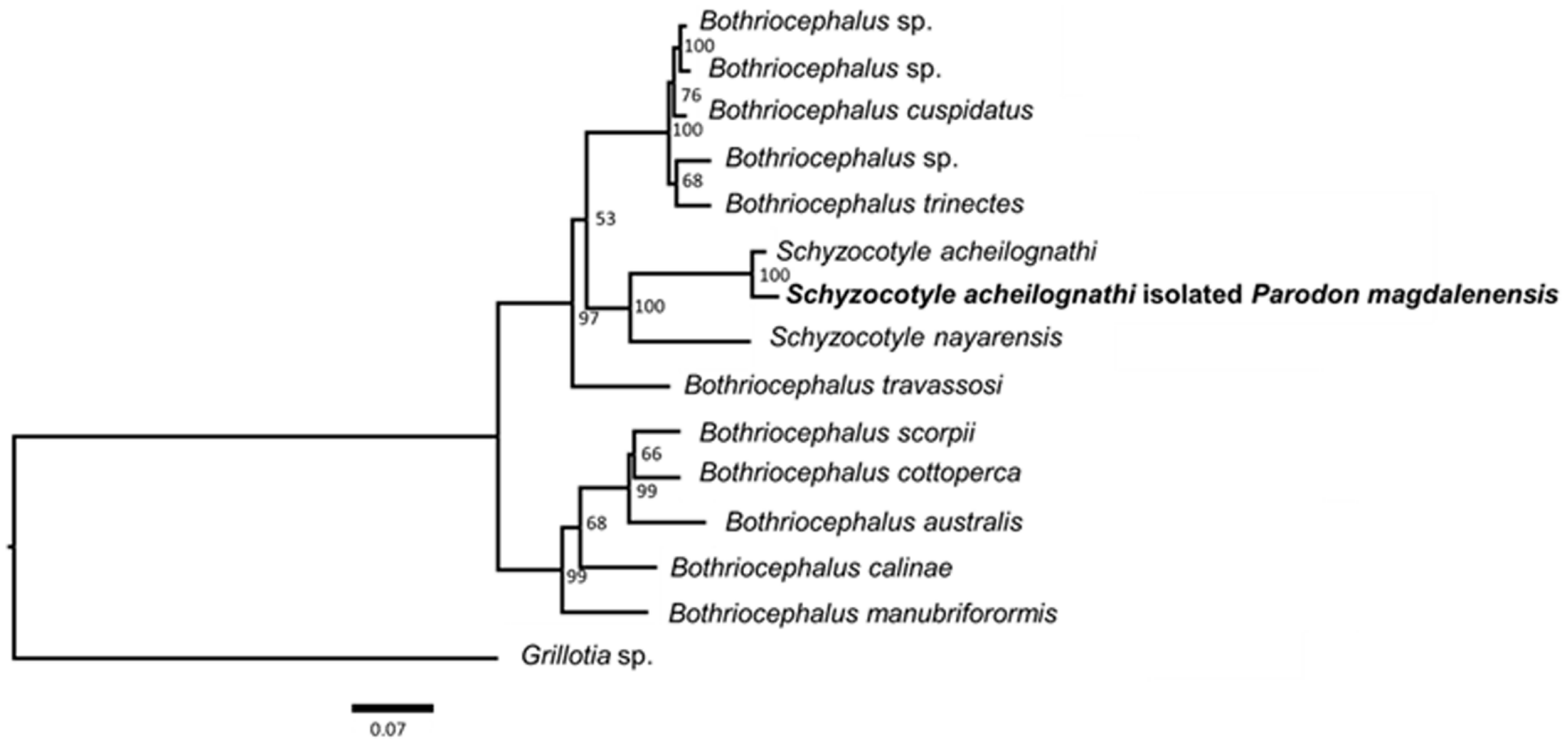
Maximum likelihood (ML) phylogram of *Schyzocotyle acheilognathi* isolated from *Parodon magdalenensis,* based on the analysis of the concatenated dataset of the three genes (16S RNA, 18S RNA y, and 28S RNA). The best-fitting substitution model was TIMe+G4. Nodal support values were estimated by running 5,000 replicates and standard non-parametric Bootstrap support (1,000 repetitions).

The isolated strobila proglottids of the cestodes collected from *Parodon magdalenensis* were molecularly characterized. The topology recovered from the analysis of the concatenated three gene dataset comprising sequences from each gene (ssrDNA:small subunit 18S nuclear rDNA; lsrDNA: partial large subunit 28S nuclear rDNA; and rrnL: partial large subunit 16S mitochondrial rDNA)—is presented (Figure 5).

## 4 Discussion

Colombia is home to a rich diversity of freshwater fish, but their conservation is threatened by multiple factors, including deforestation, pollution, and mining activities. In addition, the introduction of exotic/alien species (neozoans) has further exacerbated the situation (45). These species can significantly affect native fish through direct competition, predation, and habitat alteration. They can also influence disease dynamics (39) by introducing new parasites and pathogens (46). This is the case with *Cyprinus carpio*, a species that, in addition to altering the ecological characteristics of aquatic ecosystems (47, 48), is considered the primary vector responsible for the global spread of the parasite *S. acheilognathi* from Asia to the rest of the world. Furthermore, its presence in Colombia, where it is considered one of the most abundant invasive species in the region (49), especially in the Grande River, a tributary of the Porce River, may have contributed to the introduction of *S. acheilognathi* into the country.

The detection rate of *S. acheilognathi* in the Porce River basin is particularly alarming. This river flows 247 km northward before merging with the Nechí River, the Cauca River, and the Magdalena River, which ultimately empties into the Caribbean Sea. The Magdalena-Cauca basin is the most representative river system in the northern Andes, with 238 recorded fish species (50). At a global level, it hosts a high degree of ichthyofaunistic endemism, driven by the diverse climatic conditions to which these species have adapted (51). As the main axis of social, economic, and industrial development in the country, this basin faces multiple pressures that impact both the conservation of aquatic ecosystems and fish populations (52). In addition, the first record of the Asian tapeworm (*S. acheilognathi*) in the Porce River basin poses an additional threat to native species. Considering that *S. acheilognathi* has spread rapidly, it is highly likely that this parasite will disperse from the Porce River to the entire Magdalena-Cauca basin, thereby exposing fish to a pathogen known for its high transmission rate and adaptability to different thermal zones and hosts. Furthermore, *S. acheilognathi* is recognized as an important pathogen in aquaculture, capable of causing mortality in farmed species, making it a potential threat to the health of native ichthyofauna in the Magdalena-Cauca basin. The closest report of *S. acheilognathi* to Colombia is from Panama, supported by morphological evidence and sequence data from the ITS-1 region of the rRNA genome. The AFT case report was conducted on two species of cichlid fish, *Aequidens coeruleopunctatus* and *Cryptoheros panamensi*. Although these findings represent the geographically closest reports of *S. acheilognathi* to Colombia, transmission to the Porce River basin, where it was detected in Colombia, is not feasible due to the geographic isolation between the two basins. Therefore, direct transmission from Panama to Colombia is not possible.

Accurately identifying *S. acheilognathi* has historically been challenging, since some genera within the family Bothriocephalidae share morphological traits, leading to frequent misclassification of species from different genera and the assignment of incorrect scientific names. However, advancements in research and molecular parasitological identification techniques now allow for more precise verification of these findings. In this study, genetic sequences that showed high similarity percentages with previously reported *S. acheilognathi* sequences were obtained (8, 53), corresponding to the following markers: ssrDNA, small subunit (18S) nuclear rDNA with 98.55% similarity; lsrDNA, partial large subunit (28S) nuclear rDNA with 99.94% similarity; and *rrn*L, partial large subunit (16S) mitochondrial rDNA with 99.93% similarity. These genetic sequences were analyzed to confirm the identification of *S. acheilognathi.* As a result, this research presents the first published sequences of *S. acheilognathi* from Colombia.

By combining both morphological and molecular evidence, this study documents the first record of the invasive parasite *S. acheilognathi* in Colombia and its initial detection in the native fish species *Parodon magdalenensis*. Although this species is currently classified as Least Concern on the IUCN Red List, the potential threat posed by *S. acheilognathi* highlights the need to integrate parasitological data into conservation policies for this species. Future research should focus on mapping the geographic distribution of *S. acheilognathi* in Colombia, assessing the genetic variability of its populations across different hosts and locations, and evaluating its impact on the health of native species.

## Data Availability

The datasets presented in this study can be found in online repositories. The names of the repository/repositories and accession number(s) can be found in the article/Supplementary material.

## Footnotes

^1^ http://www.ncbi.nlm.nih.gov/BLAST
