# Supplementary material for: First report of the Asian tapeworm Schyzocotyle acheilognathi in the Colombian freshwater fish Parodon magdalenensis
Source: Front Vet Sci. 2025 Jul 9;12:1614531. doi: 10.3389/fvets.2025.1614531 (PMC12285530; doi:10.3389/fvets.2025.1614531)
Supplement: Supplementary file 1 [file Table_1.DOCX]

**Supplementary Material**

**Table 1:** Sampling locations and number of specimens.

| Localities | Longitude | Latitude | Elevation | Number of samples |
| --- | --- | --- | --- | --- |
| Porce river | -75.175 | 6.8365 | 715,0 | 47 |
|  | -75.135 | 6.9421 | 578,5 | 5 |
|  | -75.096 | 6.9605 | 537,1 | 2 |
| Guadalupe river | -75.191 | 6.8386 | 699,0 | 34 |
|  | -75.202 | 6.8271 | 772,0 | 2 |
| El Saino stream | -75.174 | 6.8991 | 719,3 | 3 |
| Santa Gertrudis stream | -75.178 | 6.8887 | 1142,1 | 2 |
| Porce III reservoir | -75.178 | 6.8887 | 720,6,0 | 8 |
|  |  |  | Total | 103 |

**Table 2:** List of genus *Bothriocephalus* sp, and *Schyzochotyle* sp. sequences included in the phylogenetic analysis with the information on DNA markers, GenBank accession numbers and host.

| **Cestodes specie** | **Host Species** | **GenBank Accession number** | | |
| --- | --- | --- | --- | --- |
|  |  | ssrDNA | lsrDNA | rrnL |
| *Schyzocotyle acheilognathi n. comb.* | *Parodon magdalensis* | PQ516284 | PQ528318 | PQ530045 |
| *Bothriocephalus australis* | *Platycephalus aurimaculatus* | KR780930 | KR780886 | KR780841 |
| *Botriocephalus celinae* | *Cephalopholis aurantia* | KR780968 | KR780921 | - |
| *Botriocephalus claviceps* | *Trinectes maculatus* | KR780957 | KR780910 | KR780871 |
| *Botriocephalus cuspidatus* | *Sander vitrus* | KR780955 | KR780908 | KR780869 |
| *Botriocephalus manubriformis* | *Istiophorus platypterus* | *-* | KR780887 | - |
| *Botriocephalus scorpii* | *Myoxocephalus scorpius* | AJ228776 | AF286942 | KR780840 |
| *Botriocephalus timii* | *Cottoperca gobio* | KR780929 | KR780885 | KR780839 |
| *Botriocephalus travassosi* | *Anguilla marmorata* | KR780959 | KR780912 | KR780872 |
| *Botriocephalus solinosomum* | *Centrolophus niger* | KR780943 | KR780896 | KR780855 |
| *Botriocephalus* sp. | *Lepomis gibbosus* | KR780953 | KR780906 | KR780867 |
| *Botriocephalus* sp. | *Lepisosteus oculatus* | KR780954 | KR780907 | KR780868 |
| *Botriocephalus* sp. | *Micropterus dolomieu* | KR780952 | KR780905 | KR780866 |
| *Schyzocotyle acheilognathi* | *Cyprinus carpio* | KR780932 | KR780889 | KR780844 |
| *Schyzocotyle nayarensis* | *Barillus* sp. | KR780969 | KR780922 | KR780878 |
| *Grillotia* sp. | *Raja radiata* | AJ228781 | AF286967 | EF103924 |
